# Longitudinal Changes in Cognition, Behaviours, and Functional Abilities in the Three Main Variants of Primary Progressive Aphasia: A Literature Review

**DOI:** 10.3390/brainsci11091209

**Published:** 2021-09-14

**Authors:** Justine de la Sablonnière, Maud Tastevin, Monica Lavoie, Robert Laforce

**Affiliations:** Clinique Interdisciplinaire de Mémoire, Département des Sciences Neurologiques du CHU de Québec, Faculté de Médecine, Université Laval, Quebec City, QC G1J 1Z4, Canada; justine.de-la-sablonniere.1@ulaval.ca (J.d.l.S.); maud.tastevin.1@ulaval.ca (M.T.); monica.lavoie.1@ulaval.ca (M.L.)

**Keywords:** primary progressive aphasia, natural history, longitudinal assessment, cognitive changes, behavioural and psychological symptoms of dementia, level of functioning

## Abstract

Primary progressive aphasias (PPAs) are a group of neurodegenerative diseases presenting with insidious and relentless language impairment. Three main PPA variants have been described: the non-fluent/agrammatic variant (nfvPPA), the semantic variant (svPPA), and the logopenic variant (lvPPA). At the time of diagnosis, patients and their families’ main question pertains to prognosis and evolution, but very few data exist to support clinicians’ claims. The objective of this study was to review the current literature on the longitudinal changes in cognition, behaviours, and functional abilities in the three main PPA variants. A comprehensive review was undertaken via a search on PUBMED and EMBASE. Two authors independently reviewed a total of 65 full-text records for eligibility. A total of 14 group studies and one meta-analysis were included. Among these, eight studies included all three PPA variants. Eight studies were prospective, and the follow-up duration was between one and five years. Overall, svPPA patients showed more behavioural disturbances both at baseline and over the course of the disease. Patients with lvPPA showed a worse cognitive decline, especially in episodic memory, and faster progression to dementia. Finally, patients with nfvPPA showed the most significant losses in language production and functional abilities. Data regarding the prodromal and last stages of PPA are still missing and studies with a longer follow-up observation period are needed.

## 1. Introduction

Primary progressive aphasias (PPA) are a group of neurodegenerative diseases that present with an insidious, progressive, and isolated impairment in language. Other cognitive functions are typically preserved for at least two years after the onset of the disease [1]. Mesulam (1982) was the first to describe six cases of progressive aphasia without accompanying signs of dementia and associated with focal perisylvian left atrophy [2]. A few years later, Snowden et al. (1989) introduced the term “semantic dementia” referring to dementia with profound loss of conceptual knowledge [3]. Afterward, Neary et al. (1998) published diagnostic criteria for progressive non-fluent aphasia and semantic dementia [4], and in 2004, a third type of PPA was described—the logopenic variant primary progressive aphasia [5]. More recently, diagnostic criteria for three main variants of PPA have been identified by Gorno-Tempini et al. (2011) [6]. The classification is based on language features and can be supported by the pattern of atrophy found on neuroimaging and pathological examination. We used the classification from Gorno-Tempini et al. (2011) as a framework for this study, but it is noteworthy that other clinical diagnoses and mixed cases exist even if not in the scope of this review (e.g., primary progressive apraxia of speech [7]).

According to the criteria of Gorno-Tempini et al. (2011), the non-fluent/agrammatic variant (nfvPPA) is characterized by the presence of agrammatism and/or apraxia of speech. Patients can also present with impaired comprehension of syntactically complex sentences but typically have spared single-word comprehension and object knowledge. Structural neuroimaging shows prominent cerebral atrophy in the left posterior frontoinsular region. This variant is most often associated with tau pathology [8] and classified as frontotemporal lobar degeneration (FTLD). The semantic variant (svPPA) features impaired single-word comprehension and confrontation naming. Patients can also show surface dyslexia or dysgraphia (i.e., reading or writing from sounds) and impaired object knowledge, especially for items that are less frequent or familiar to them (e.g., apple vs. mango). Brain imaging shows atrophy in the anterior temporal regions bilaterally but predominantly in the left hemisphere. Its underlying pathology is predominantly TDP-43 [8], and this variant is also considered as part of the FTLD spectrum. Finally, the logopenic variant (lvPPA) is associated with altered repetition of long sentences, single-word retrieval difficulties, and phonologic errors (e.g., apple–papple). Atrophy is predominant in the left posterior perisylvian or parietal regions. Neuropathology is predominantly amyloid-β [8] and consequently, lvPPA is classified as a variant of Alzheimer’s disease.

Following the criteria by Gorno-Tempini et al. (2011), several authors sought to improve the characterization of the PPA variants to improve diagnostic accuracy [9]. Indeed, Perry et al. (2019) reported svPPA and nfvPPA diagnosis to be highly stable, with only rare diagnosis changes through follow-up [10]. Regarding intervention, the therapeutic arsenal of the clinician consists mostly of speech–language therapy [11,12], and pharmacotherapy such as neuroleptics for the management of BPSD. In lvPPA, a recent study suggested that the use of cholinesterase inhibitors was justified for patients with an underlying Alzheimer’s pathology [13].

At the time of diagnosis, patients and their families often inquire about what to expect in terms of the progression of symptoms and nature of upcoming deficits, which will have a significant impact on their daily life and functional communication. Few studies have explored the challenges faced by patients and caregivers [14,15,16]. Greater knowledge of the evolution of the three PPA variants would allow better counseling and help orient better clinical approaches for this population as the disease progresses. For example, it would help identify specific targets for intervention approaches that have been found to carry significant changes for PPA patients and their caregivers such as functional communication intervention [17,18,19,20], as well as education and support groups [21,22]. Although there is a growing interest in these pathologies, as proven by the increasing number of publications in the literature, there remains few available data on the longitudinal changes of PPAs. Studies on the evolution of language, cognition, level of functioning, and behavioural changes are scarce and have been hindered by small sample sizes. To our knowledge, no review on PPA evolution has been published yet. Therefore, results from the various studies published have not been put together to highlight tendencies for PPA in general but also for each variant specifically. The aim of this work was, therefore, to review the current literature on longitudinal changes occurring in patients with PPA. More specifically, the objective was to draw conclusions from the existing literature for each variant regarding cognition, language, BPSD, and functional abilities. Our hypothesis was that the type and magnitude of longitudinal changes across these elements would differ in each PPA variant, therefore displaying tendencies and profiles and allowing better counselling for patients and their families.

## 2. Materials and Methods

A comprehensive review of the literature was undertaken in PubMed and Embase databases to identify previous studies on the evolution of PPAs. The initial search was conducted from October 2020 to May 2021. The search terms used were “primary progressive aphasia”, “aphasias, primary progressive”, “primary progressive aphasias”, “progressive aphasia, primary”, “progressive aphasias, primary”, “epidemiology”, and “natural history”. An updated search was conducted in August 2021 with the same procedure. In addition to all of the terms mentioned above, the following search terms were also included: “non-fluent variant PPA”, “nfvPPA”, “logopenic variant PPA”, “lvPPA”, “semantic variant PPA”, “svPPA”, “frontotemporal dementia”, “progression”, “decline”, “history”, and “mortality”.

No restrictions were made regarding the language in which the articles were written. Studies included met the following criteria:

Study design: meta-analysis, prospective or retrospective studies, comparative or not with other groups (healthy control or other neurodegenerative diseases);

Participants: all patients with a clinical diagnosis of PPA according to the Gorno-Tempini et al. (2011) criteria;

Outcomes measures: all clinical data on which assessment was based validated scales or consensus clinical criteria.

Case reports, studies focusing only on paraclinical measurement (neuroimaging or biomarkers), and studies with no follow-up available were excluded from the review, as were studies published in journals with impact factors of less than two.

One author read all the titles and abstracts of database records and selected articles that corresponded to the selection criteria mentioned above. Subsequently, two authors independently reviewed the full-text records and verified if selection criteria were still met. Disagreements were resolved by consensus discussion. Eligible manuscripts were then independently reviewed by two of the authors. In addition, the references cited in the articles were screened to look for additional references that might not have been identified in the initial literature search. The following data were extracted: first author name, date of publication, impact factor, study design, study country, sample size, number of included subjects and diagnosis, type of clinical assessment, follow-up time, and main outcomes. Clinical assessment was divided into general cognition, language, behavioural and psychological symptoms of dementia, and level of functioning.

## 3. Results

**Figure 1:**
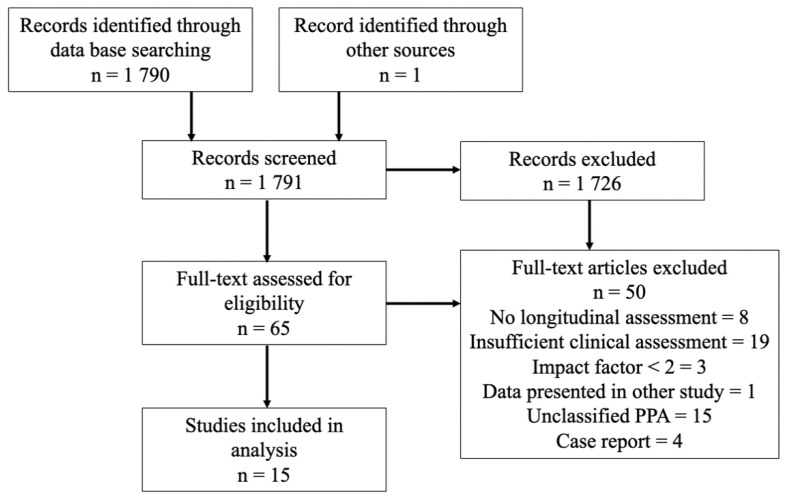
Flowchart of the selection procedure.

As of August 2021, approximately 1790 articles were published on PPA in PubMed and Embase databases. According to our search paradigm, and after removing duplicate records, 65 texts were assessed for eligibility. A total of 15 studies were included, as shown in Figure 1. In total, 14 consisted of observational studies and 1 was a meta-analysis [23]. Studies published before 2011, therefore not based on consensus criteria by Gorno-Tempini et al. (2011), were excluded. All 14 observational studies taking into account at least one PPA variant are summarized in Table 1. The meta-analysis is discussed below.

Altogether, the 14 observational studies comprised 745 patients who were classified as follows: nfvPPA (277), svPPA (281), and lvPPA (172). One study included 13 patients that had either nfvPPA or svPPA variants. Another study included 2 patients with unclassified PPA in their group study of 35 patients with PPA [28]. Mixed variants were not analyzed. Eight studies included all three PPA variants, representing 470 patients divided as follows: 149 lvPPA, 150 nfvPPA, and 171 svPPA. Five studies included a control (HC) group for a total of 156 healthy controls (see Table 1). Four studies focused only on FTLD variants, one study included only one variant of PPA (10 lvPPA), and in five studies, a clinical group was added that is Alzheimer’s disease (AD group) and/or behavioural frontotemporal lobar degeneration (FTLD).

These fourteen observational studies were conducted in Canada (one), Italy (two), Spain (two), the United States (two), Australia (five, four of which came from the same database Frontier), the Netherlands (one), and Japan (one). Eight of them were performed in a prospective fashion. The most recent prospective study published by Foxe et al. (2021) also included the most complete and largest sample of PPA patients with 44 nfvPPA, 62 svPPA, and 41 lvPPA. Cosseddu et al. (2020) and Gómez-Tortosa et al. (2016) studied retrospectively the largest samples of PPA patients, (respectively, 77 nfvPPA and 40 svPPA; 39 nfvPPA and 41 svPPA) [29,35].

Patients’ mean age at baseline ranged from 58 to 70 years, with most patients being in their mid-60s at initial assessment. There was a tendency towards more female patients with 9 out of 14 studies having 50% or fewer male patients (although one study [30] had 72% male patients). The mean duration of symptoms from onset to initial assessment ranged from 2.6 to 6.7 years, with the majority between 3 to 4 years.

In most group studies, patients were assessed yearly, for a year or two. Only three studies had a mean follow-up of five years or more. Clinical aspects assessed and evaluation tools were very heterogeneous among studies. Only one study analyzed mortality data [28].

**Figure 2:**
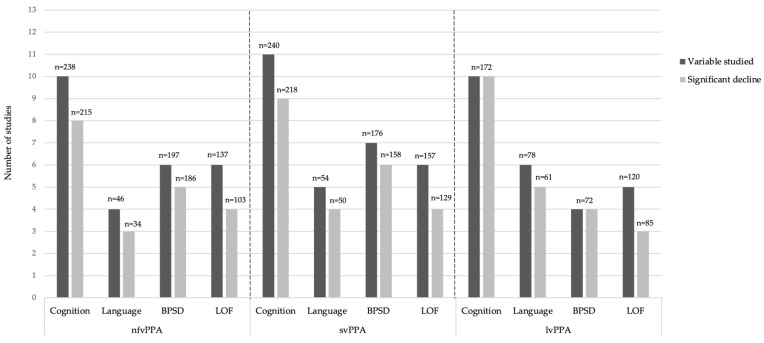
Summary of findings in 13 of the observational studies included.

Figure 2 shows a visual representation of the numbers of studies, and the number of patients they included, which evaluated cognition, language, BPSD, or functional abilities, and the number in which a significant decline was reported. It is important to note however that the designs of the studies were very heterogeneous. Some of them had very precise hypotheses and sometimes, changes in clinical scores over time were not available in the publication, nor in its supplementary material, which prevented us from extracting the data. Notably, the study from Linds et al. (2015) was excluded from the graph. In this publication, 13 patients with nfvPPA or svPPA were included, but the number of patients belonging to each variant was not precise. Therefore, Figure 2 comprises the 13 other observational studies.

### Clinical Assessment

All included observational studies used validated international scales to evaluate the different aspects of cognition, language, autonomy, and behavioural and psychiatric symptoms of dementia (BPSD). Cognition was the most frequently assessed clinical aspect and was included in 12 studies. The Addenbrooke’s Cognitive Examination (ACE) and its subsequent versions (III and revised) [38,39], as well as the Mini-Mental State Examination (MMSE) [40], were the most frequently used tools, in six and five studies, respectively. Other cognitive evaluation tools used were the Clinical Dementia Rating Scale (CDR) and its modified version for Frontotemporal Lobar Degeneration (FTLD-CDR) in four studies [41,42]. Some of the less frequently used tools were the Cambridge Behavioural Inventory-Revised (CBI-R) [43] and the Wechsler Adult Intelligence Scale (WAIS) [44]. Some subtests of these different neuropsychological batteries were also used individually. Although language was invariably evaluated at baseline, only five studies reported longitudinal assessment of language. The Boston Naming Test (BNT) was the most frequently used test, in four studies [45]. The Progressive Aphasia Severity Scale (PASS) [46] was used in two studies. The other tests were used in only one study each and are presented in Table 1. Language domains assessed varied across studies and included phonemic and semantic fluency, confrontation naming, comprehension, reading, writing, and repetition.

Eight studies evaluated the onset and evolution of BPSD. The most frequently used test was the Neuropsychiatric Inventory (NPI), a semi-structured clinician interview of caretakers [47] in five articles. The Cambridge Behavioural Inventory-Revised (CBI-R) [43] and the Frontal Behavioural Inventory (FBI) [48] were both used in two studies each. Level of functioning in basic activities of daily living (BADL) and instrumental activities of daily living (IADL) were assessed in seven studies, directly or through clinical questionnaires. The most frequently used tool was the disability assessment for dementia (DAD) [49], in three studies. One study used the Functional Activities Questionnaire (FAQ) [50], which was recently proven to be a useful functional measure for longitudinal changes in FTD [51].

Some studies also looked at other variables not included in the scope of this review such as neuroimaging patterns and progression of atrophy, development of parkinsonian syndromes, genetics, and pharmacotherapy.

## 4. Discussion

We proposed herein the first comprehensive review of longitudinal changes in cognition, language, BPSD, and functional abilities in PPA. A total of 14 observational studies were included in this review, as well as a meta-analysis studying survival. General findings of PPAs will first be discussed, followed by specific findings for each variant and then survival data. Finally, limitations of the current work and the impact of the findings on clinical care and future perspectives will be addressed.

### 4.1. Similarities between All Three Main Variants of PPA

In all three PPA variants, studies that assessed cognition reported a decline over time. Previous studies have indeed demonstrated that even if language is primarily affected in PPA, other cognitive functions are impaired as well [5,52,53,54]. Regardless of the variant, two studies described a faster decline in cognition for PPAs, when compared to AD [25,34]. Indeed, Funayama et al. (2019) reported an annual rate change in the CDR sum of boxes of 3.4 ± 1.1 in their group of 10 lvPPA patients. This is a greater rate of decline than what Doody et al. (2010) previously reported in their group of 597 AD patients [55]. In Hsieh et al. (2012), the annualized rate of change was greater in all three PPA variants (9 lvPPA, 12 nfvPPA, and 17 svPPA patients) when compared to the AD group (17 patients) on the ACE-R. Over a year, the PPA patients lost on average 10 points, as compared to less than 5 by AD patients. However, these findings must be interpreted with caution since several neurocognitive tests are influenced by language abilities. Clinically, it is common to see PPA patients with lower scores on cognitive testing that do not correspond to the level of functioning on collateral history. Therefore, specific assessment and neuropsychological tools that take into account language impairments should be used with PPA patients [56]. Only one study included in this review explored the correlation between dementia progression and decline in language [32], but no such association was found. On the other hand, Funayama et al. (2019) did describe a relationship between dementia progression and language decline, although no statistical analysis was performed.

Among the few studies which evaluated changes in language, a decline was described in all variants. Ferrari et al. (2019) reported mutism in 31% of patients at 2.7 years. Although the MMSE score and fluent language at baseline were previously described as protective factors for mutism [57], these relationships were not established in the study by Ferrari et al. (2019).

There were discordant findings among the studies regarding BPSD. One study described no influence of behavioural and psychiatric symptoms at baseline on disease progression [33]. Conversely, another study found that apathy and stereotypical behaviour at baseline were predictors of functional decline for nfvPPA [30]. Linds et al. (2015) showed that education was a protective factor for disinhibition, but this finding was not indicated in other publications [27]. The main limitation regarding BPSD is the heterogeneity of the symptoms, and NPI is often limited in its description. Moreover, BPSD fluctuation over time could complicate the interpretation of the results. BPSD assessment would require a longer follow-up, with several validated scales and consideration of qualitative data for a more exhaustive list of symptoms [58].

Regarding the level of functioning in IADL and BADL, all three variants showed a decline at follow-up. In Foxe et al. (2021), over a period of four years, the mean DAD total scores with 95% confidence intervals, decreased from 82.7 (76.1–89.3) to 48 (39.5–56.4) for lvPPA patients, from 86 (79.4–92.5) to 51.3 (43.4–59) for nfvPPA patients, and from 85 (79.8–90.1) to 55.6 (50.3–60.9) in svPPA patients. One study demonstrated a direct link between the decline in functioning and cognition, with the relationship increasing over time [36]. This link was already described in a previous study of 2009 studying changes in functioning and cognition in 9 nfvPPA and 11 svPPA patients [59]. Moreover, cognition at baseline and its deterioration in the first year were predictive factors of greater functional incapacities throughout the course of the disease [30]. This is coherent with two previous studies; one showed that a higher MMSE score at baseline was a predictor of preservation of autonomy in the following years [57], while the other, conversely, correlated lower MMSE and FTLD-CDR scores at baseline with more rapid change overtime in functional measures for nfvPPA and svPPA, respectively [51]. This finding is not surprising and could be explained by the fact that patients with lower functional and cognitive scores at baseline have either a more aggressive disease or a lower cognitive reserve [60,61]. Education also seemed to be a protective factor for functional impairment [28,33]. Indeed, patients with higher education are most likely to have a higher cognitive reserve and therefore to be able to compensate longer in IADL and BADL. In their study, Ferrari et al. (2019) reported a severe functional dependency in 20% of the patients at 2.5 years. Other data from the literature reported a need for assistance in BADL in 50% of the patients at five years [57]. This is in contrast with the findings of O’Connor et al. (2016), who described a sparing of functional abilities for five years from onset. However, in this study, which included only nfvPPA and svPPA patients, only one tool was used for assessment of functioning, and one could argue that more extensive deficits would have been detected with a more comprehensive evaluation.

### 4.2. Non-Fluent Variant of Primary Progressive Aphasia

Four studies revealed that nfvPPA patients showed a greater decline in language production over time. In Ash et al. (2019), the decline in language production was more important for fluency and grammar, whereas in Rogalski et al. (2011), participants showed a decline in all language domains, with each of the three patients being too impaired to complete at least one of the different measures [24,32]. Similarly, Ulugut et al. (2021) found that out of eight patients who displayed mutism at follow-up, seven of them were classified as nfvPPA variant [37]. Finally, in Foxe et al. (2021), these patients showed a disproportionate impairment in verbal fluency at all time points and a faster decline in language during follow-up [36]. This decline in speech production is explained by the progression of the atrophy in the left frontal and subcortical areas, regions that are important networks for language production [62,63,64]. These findings are coherent with a previous study in which the nfvPPA patients were not able to complete ACE-R at one year of follow-up, due to language deterioration [59].

Regarding behavioural and psychiatric symptoms, nfvPPA patients have a tendency towards negative symptoms [36,59]. Indeed, they showed a higher frequency of depression and a greater need for antidepressants, as opposed to antipsychotics [29]. In their study, Van Langenhove et al. (2016) reported that apathy was the most prominent symptom, present in 46% of nfvPPA patients at baseline and increased to 68% at follow-up.

There also seems to be a tendency in this variant for a faster decline in the level of functioning. Hsieh et al. (2012) reported a faster decline at the Frontotemporal Dementia Rating Scale (FRS), an assessment tool measuring, among others, changes in everyday abilities such as using the phone and taking medication, in the nfvPPA group than in AD patients. Indeed, over 12 months, among the group of 12 nfvPPA patients, over 80% of patients showed a decline in the FRS. Similarly, in Foxe et al. (2021), nfvPPA patients had a faster decline in the level of functioning, compared to svPPA, with an annual rate of decline on the DAD total score of 8.7 points, compared to 7.4 points. Another study revealed that nfvPPA and lvPPA had a worse decline in daily life activities at a one-year follow-up, with the first group having the greatest impairments in self-care [31]. In line with these findings, Mioshi et al. (2009) had previously reported that nfvPPA patients showed significant changes both in BADL and IADL at follow-up [59].

### 4.3. Semantic Variant of Primary Progressive Aphasia

Specific findings regarding BPSD were highlighted in svPPA patients. First, they tended to show behavioural symptoms earlier in the disease course and more frequently, compared to the other variants, as well as AD and behavioural variant of frontotemporal dementia (bvFTD) [28,33,37]. Indeed, Van Langenhove et al. (2016) found that 74% of svPPA patients had behavioural changes at baseline, compared to 54% of nfvPPA patients and 47% lvPPA patients. At follow-up, the tendency remained with 80% of svPPA patients showing at least one behavioural symptom. In the study by Matias-Guiu et al. (2015), half of the svPPA patients (two out of four) developed behavioural disorders. The most frequent disturbances were stereotypical behaviour, empathy loss, and apathy. These findings are consistent with the study by O’Connor et al. (2016), which included 18 svPPA patients and found that patients with this variant displayed more stereotypical behaviour at baseline (60% vs. 9% in nfvPPA). Moreover, in Ulugut et al. (2021), 58% of svPPA patients (group of 24) eventually met diagnostic criteria for bvFTD. Compared to nfvPPA, svPPA patients also showed a higher frequency of agitation and delirium/hallucinations [29]. There was a significant difference in the severity of irritability, agitation, delirium, and apathy and a greater need for antipsychotic drugs. Increased behavioural dysfunctions in svPPA, especially disinhibition, were already underlined in the literature [65,66]. Heterogeneity in the results could, in part, be explained by a misdiagnosis of the right temporal variant of FTLD, also called the right semantic variant. It is possible that the course of the disease in the left svPPA and right semantic variant could be significantly different. A recent study showed that prosopagnosia, episodic memory impairment, and behavioural changes such as disinhibition, apathy, compulsiveness, and loss of empathy were the most common initial symptoms for the right temporal variant, whereas, during the disease course, patients developed language problems such as word-finding difficulties and anomia [67]. Distinctive symptoms of the right semantic variant, compared to the other groups, included depression, somatic complaints, motor, and mental slowness.

Interestingly, a few studies suggested that svPPA patients had a longer duration of symptoms before the diagnosis. In Van Langenhove et al. (2016), symptoms duration at baseline was 4.4 years for svPPA, compared with 2.3 and 3.5 years in nfvPPA and lvPPA, respectively. In Hseish et al. (2012), svPPA patients had a mean disease duration of 4.2 years at the time of diagnosis, compared with 2.3 and 3.9 years for nfvPPA and lvPPA. This tendency for a longer duration of symptoms in the svPPA variant, although not always statistically significant, was also reported in other studies [66,68]. This could be explained by the fact that the loss of semantic knowledge can be masked by word-finding difficulties (e.g., vague words, circumlocutions) and therefore may be overlooked by family members, which, in turn, delays recognition of the syndrome. In contrast, nfvPPA has a more striking presentation with agrammatism and halting speech. Furthermore, lvPPA variants present with impaired single-word retrieval in spontaneous speech, which is more frequently recognized by family members as an early sign of dementia.

### 4.4. Logopenic Variant of Primary Progressive Aphasia

Five studies revealed that the lvPPA patients showed a worse decline in global cognition, compared to other variants [26,28,31,36,37]. Foxe et al. (2021) found that lvPPA patients had a twice as rapid decline rate in overall cognition, despite performing intermediate to the other variants at baseline. Funayama et al. (2019) also showed this tendency to faster progression. In their study, lvPPA patients evaluated with the clinical dementia rating sum of boxes, had a change in dementia severity every 1.7 years and reached severe dementia (CDR 3) in 7.3 ± 1.6 years, a faster progression, compared to Alzheimer’s disease [55]. In Ulugut et al. (2021), 83% of lvPPA patients (group of 18) acquired global cognitive impairment consistent with Alzheimer’s disease dementia. Moreover, among cognitive skills, memory seemed to be the most frequently and severely affected ability, as demonstrated in four studies [28,31,36,37]. The 10 lvPPA patients in the Funayama et al. study (2019) showed episodic memory deficits beginning at 4.0 ± 2.0 years after onset. These findings are not surprising considering that the logopenic variant is most frequently associated with Alzheimer’s pathology [8,69]. Studies that included thorough imaging analysis also showed a greater cognitive decline in lvPPA, associated with progression of brain atrophy in the regions typically damaged in AD [62]. Level of functioning and BPSD were less studied in lvPPA than in the two other variants. Foxe et al. (2021) found a faster rate of decline in the level of functioning in lvPPA in comparison to svPPA with an annual rate of decline on the DAD total score of 8.7 and 7.4 points, respectively, within their groups of 41 lvPPA and 62 svPPA. As for BPSD, they were found to be less prevalent in lvPPA than in the two other variants, with apathy being the most frequent [31].

### 4.5. Survival Data

The meta-analysis by Kansal et al. (2016) was the only study, to our knowledge, which addressed survival in PPA. In total, 27 studies focusing on survival and years of life lost (YLL) were included with patients presenting AD, corticobasal degeneration (CBD), progressive supranuclear palsy (PSP), and all FTLD variants (svPPA, nfvPPA, bvFTLD, and FTLD-ALS). In contrast to survival, which emphasizes life expectancy, YLL highlights premature mortality. YLL is, therefore, useful for quantifying premature deaths in policy contexts. The median survival in the svPPA variant was significantly longer than in nfvPPA (12 years versus 7.66 years). However, the mean survival for svPPA was estimated at 7.45 years, and 8.11 years for nfvPPA with no statistically significant difference. To explain these contradictory findings between median and mean survival findings, Kansal et al. suggested an artifact in the analysis due to heterogeneity in the included studies (sampling methods and regional context). They also raised the hypothesis that the presence of a negative or positive skew could be a statistical reflection of the survival profile. Indeed, a negative skew could reflect a young- to mid-life onset disease with a sufficiently long course and few premature deaths. A positive skew would be more likely associated with a disease characterized by a very short course, as the outliers are those with unusually long survival. Patients with nfvPPA had the longest mean survival between all the neurodegenerative diseases with a significant difference, compared to the PSP and CBD groups. Mean and median YLL estimated from survival (years) were, respectively, estimated to be 10.54 years and 8.97 years for nfvPPA, and 13.56 and 7.71 years for svPPA. The main limitations of this study were the heterogeneity of the data, the absence of lvPPA patients, the presence of uncertain associations between clinical and sociodemographic outcomes, and contradictory results in some of the studies included. Moreover, they did not examine the effects of confounding features and comorbid conditions. Causes of death were not reported either. Indeed, it would be helpful to know if death occurred before the final stages because of intercurrent medical conditions.

In this special issue, our group has provided recent insights into survival in the three PPAs. Indeed, significant differences in survival were found with svPPA showing the longest and nfvPPA showing more neurologically-related causes of death [70].

### 4.6. Limitations

Studies in the current review included were very heterogeneous, with none assessing longitudinal changes in cognition, language, BSPD, and functioning altogether. In fact, only six of them studied at least three of our outcomes of interest. Moreover, these clinical aspects were mostly assessed through different assessment tools (e.g., MMSE, ACE-R) and assessments by specialists such as speech–language pathologists or neuropsychologists were uncommon. Duration of follow-up was relatively short (only one or two years after diagnosis), and very few patients were followed until death. All these elements prevented us from extracting solid data about the long-term outcomes of PPA patients. Moreover, PPA being a rare type of dementia, sample sizes were relatively small, with a mean of 21 patients per variant among the studies. The lvPPA variant was also underrepresented, especially since five studies included only FTD variants (svPPA and nfvPPA). This underrepresentation of lvPPA patients could be explained by the fact that lvPPA is the most recently described variant. Indeed, no survival data were available for this variant and epidemiological information remains lacking.

The research method used in the present review also has its limitations. First, only two databases were explored, and restrictive inclusion criteria were used. Moreover, no assessment of the methodological quality of each study was performed. A systematic review and meta-analysis conducted according to the Preferred Reporting Items for Systematic Reviews and Meta-Analyses statement (PRISMA) [71] could contribute to additional and more significant outcomes. Indeed, clinical assessments of at least 745 PPA patients are currently available, with 172 lvPPA, 277 nfvPPA, 281 svPPA, and 15 unclassified PPAs. However, as some studies were conducted within the same research centres, it is possible that the actual number of participants censored was lower, therefore limiting the generalization of findings. Further studies with a longer follow-up period until death remains to be conducted. It could be particularly useful to provide a more exhaustive evaluation of the end stages of PPA, causes of death, and epidemiological data. Finally, in the near future, the focus should be on lvPPA patients whose data are actually less well represented in the literature, compared to FTLD variants.

## 5. Conclusions

This study sheds further light on our current understanding of the longitudinal changes in cognition, behaviours, and functional abilities in PPA variants but, most importantly, it provides useful information for patients and their families. In addition to confirming general tendencies for the evolution of PPA, our study highlights differences in the progression of the three variants with svPPA, showing more behavioural disturbances, nfvPPA progressing towards more language and functional deficits, and lvPPA displaying a worse decline in global cognition, especially memory. These findings are also relevant to prioritizing the clinical care offered to PPA patients and their caregivers by highlighting the challenges they are most likely to face. For example, education and support groups for patients and their caregivers were demonstrated as a worthy component of PPA patients’ care and are indicated regardless of the variant [22,72]. However, referrals to groups aiming at managing behavioural disturbances are likely to be highly important for patients with svPPA and their caregivers, given the high prevalence of BPSD in this variant. The decline in the language is expected in all three variants, and therefore, referral to a speech–language pathologist can be useful. Indeed, previous studies highlighted the efficacy of speech and language interventions for PPA patients, as well as maintenance of the gains after the treatment period [11,19,73,74]. Even teletherapy proved to be beneficial in mild-to-moderate cases, therefore opening the door to new possibilities and better access to therapy [75]. Knowing that nfvPPA patients are most likely to have a faster language decline and to progress to mutism, implementation of compensatory communication tools such as assistive augmentative communication (AAC) devices should be a priority in the management of the disease and as early as possible so that the patient is still able to learn to use it. Indeed, the use of AAC devices can allow patients to maintain effective communication [12]. Moreover, clinical care of nfvPPA should include information to caregivers about the different options regarding home care given the faster decline in the level of functioning in this variant. Finally, given the likely memory impairment in lvPPA, patients and caregivers should be informed about ways to compensate for this deficit in their daily life.

This study also highlights important shortcomings in the literature and the need for more research on this subject, especially regarding the logopenic variant. Future studies should aim at better documenting cognitive and language functions, BPSD, and functioning in everyday life, throughout the disease, in order to improve management of PPA in clinical settings.

## Figures and Tables

**Table 1:** Observational studies exploring some elements of the longitudinal changes in cognition, behaviours, and functional abilities in the main PPA variants.

Authors (Year)	Participants	Study Design	Follow-Up	Clinical Assessment	Main Results
Rogalski et al. (2011) [24]	lvPPA: *n* = 6 nfvPPA: *n* = 3 svPPA: *n* = 4 HC: *n* = 27	Prospective group study	1 FU at 2 years	(a) Cognition: Clinical judgment, behavioural scales, neuropsychological tests (b) Language: NAT, PPVT, WAB-AQ, PASS, BNT (c) Imaging: MRI	(a) Initial clinical distinctive neuropsychological patterns become blurred at follow-up. (b) Persistence of differential impairment of word comprehension in svPPA and grammatical processing in nfvPPA. For lvPPA, marked decline in naming ability. (c) No correlation between loss of cortical volume and clinical progression of aphasia. Preservation of lateralization to left hemisphere.
Hsieh et al. (2012) [25]	lvPPA: *n* = 9 nfvPPA: *n* = 12 svPPA: *n* = 17 AD: *n* = 17	Retrospective group study	Two assessments at least 12 months apart	(a) Cognition: ACE-R, FRS	(a) Faster decline in PPA than in AD, but no difference between variants. Longer time between symptoms onset and clinical diagnosis for svPPA compared to nfvPPA and AD.
Leyton et al. (2013) [26]	lvPPA: *n* = 13 svPPA: *n* = 11 HC: *n* = 17	Prospective group study	Yearly Mean duration of 3 years	(a) Cognition: MMSE, ACE-R (b) Language: Confrontation naming, single-word comprehension and repetition	(a) 3x greater decline in lvPPA for ACE-R and MMSE, the most rapid decline being in attention and visuospatial domains. lvPPA: Global impairment (meeting criteria for dementia) by 12 months. svPPA: Impairments confined to verbally mediated tasks (sparing visuospatial domain) for up to 3 years. (b) Duration of symptoms had an effect on memory and naming performances, with no differences between PPA groups.
Linds et al. (2015) [27]	nfvPPA + svPPA: *n* = 13 bvFTD: *n* = 30 AD: *n* = 118	Retrospective group study	Every year	(a) BPSD: FBI, NPI, FTLD-CDR (b) LOF: FRS	(a) Education was a predictor for ROC on the FBI-disinhibition subscale. FBI total and its sub-scale scores for apathy and disinhibition correlated with duration of illness. (b) LOF only studied at baseline.
Matias-Guiu et al. (2015) [28]	lvPPA: *n* = 17 nfvPPA: *n* = 12 svPPA: *n* = 4 Unclassified: *n* = 2 HC: *n* = 16	Prospective group study	Every 4 to 6 months Mean length unknown	(a) Cognition: MMSE, ACE-R (b) Language: BNT, letter/word verbal fluency, BDAE («Cookie Theft» picture), Barcelona Test (language subtests), PASS (c) LOF: IDDD, FAQ	(a) 74.3% developed a non-language symptom or deficit (PPA-plus). (b) Median time between onset and PPA-plus = 36 months (c) nfvPPA: Parkinsonism, behavioural disorder and motor neuron disease. (d) lvPPA: Memory or global impairment. (e) svPPA: Behavioural disorder. (f) Right laterality and years of education associated with lower risk of progression to PPA-plus while lvPPA is associated with higher risk.
Gómez-Tortosa et al. (2016) [29]	nfvPPA: *n* = 39 svPPA: *n* = 41	Retrospective group study	Biannual Mean length = 5 years	(a) BPSD: NPI-Q, pharmacotherapy	(a) No differences in first behavioural assessments. (b) At last assessment: svPPA: higher frequency and intensity of agitation and higher frequency of delirium/hallucinations. Greater need for antipsychotics (*p* = 0.001), 49% of patients. nfvPPA: higher frequency of depression. Greater need for antidepressants.
O’Connor et al. (2016) [30]	nfvPPA: *n* = 11 svPPA: *n* = 18	Prospective group study	Baseline and one FU at mean 1.4 years	(a) Cognition: ACE-R (b) BPSD: CBI-R (c) LOF: DAD	(a) Greater memory impairment at baseline in svPPA. (b) More stereotypical behaviour at baseline in svPPA. (c) Similar decline in functional score in both groups. svPPA: Functional and cognitive scores at baseline are predictors of functional decline. nfvPPA: Functional score at baseline is a predictor of functional decline. Functional abilities remained virtually intact up to 5 years from disease onset while behavioural changes were present from an early stage.
Van Langenhove et al. (2016) [31]	lvPPA: *n* = 21 nfvPPA: *n* = 22 svPPA: *n* = 30 bvFTD: *n* = 33 AD: *n* = 31	Prospective group study	1 FU at a mean of 12 months	(a) Cognition: CDR, ACE-III, CBI-R (b) BPSD: CBI-R (c) LOF: CBI-R	(a) Baseline: Memory impairment lvPPA > nfvPPA. Follow-up: Memory remains less impaired for nfvPPA. (b) Baseline: Prevalence = svPPA > nfvPPA > lvPPA. In svPPA, mostly stereotypical behaviour, empathy loss and apathy. In nfvPPA and lvPPA, mostly apathy. Follow-up: >70% developed a clinically relevant change in at least one behavioural symptom. Apparition of behaviour changes in 38 to 50% patients. Hallucinations and delusions remained rare in all groups. (c) Baseline: Similar level of impairment for daily activities across PPA except for greater impairment in everyday skills in lvPPA. Follow-up: Decline in everyday skills less pronounced in svPPA.
Ash et al. (2019) [32]	lvPPA: *n* = 14 nfvPPA: *n* = 9 svPPA: *n* = 11 bvFTD: *n* = 14 HC: *n* = 36	Prospective group study	1 FU at a mean of 26 months	(a) Cognition: MMSE, FDS, RDS, (b) Language: BDAE («Cookie Theft» picture), BNT, phonemic and semantic fluency	(a) Decline in global cognition in all variants. For nfvPPA and bvFTD, significant decline on MMSE only. (b) Decline in language production over time in all variants but more so in nfvPPA. No difference in rate of decline in language between variants. No correlation between decline in cognition and language.
Ferrari et al. (2019) [33]	lvPPA: *n* = 23 nfvPPA: *n* = 26 svPPA: *n* = 19	Retrospective group study	M = 2.06 years Frequency unknown	(a) Cognition: MMSE (b) BPSD: NPI (c) LOF: BADL, IADL (d) ApoE4 status	(a) Mean loss of 4 points at 1 year and 9 at 2 years. (b) No influence of BDSP on disease progression. (c) Severe functional dependency in 20% at 2.5 years. Cognitive decline in 1st year is a risk factor for functional impairment while high education is protective. (d) Cognitive decline associated with ApoE4 status. Higher prevalence of mutism in ApoE4 patients.
Funayama et al. (2019) [34]	lvPPA: *n* = 10	Prospective group study	Every year Duration 6 to 10 years post onset	(a) Cognition: CDR (b) Language: Standard Language Test of Aphasia (c) BPSD: NM scale	(a) Decline in CDR of 3.4 points/year, change of dementia severity every 1.7 year. 4.1 year to reach CDR 1 (mild dementia), 5.7 years to DCR 2 (moderate), and 7.3 years to CDR 3 (severe). Dementia progression parallels linguistic decline. Difficulties with using electronic appliances began 3.3 years post onset, episodic memory deficits 4 years post-onset, and topographical disorientation 5.2 years. 60% could not recognize family members, 50% with pica, 30% with mirror sign (visuospatial deficits and body schema disorder).
Cosseddu et al. (2020) [35]	nfvPPA: *n* = 77 svPPA: *n* = 40 bvFTD: *n* = 286	Retrospective and prospective group study	Every year Mean length = 3.1 years	(a) Cognition: FTLD-CDR (b) BPSD: FBI, NPI	(a) Increase in negative symptoms with disease severity in bvFTD and PPA. (b) Increase in positive symptoms until intermediate phases, followed by reduction in later phases. Positive symptoms less common in nfvPPA.
Foxe et al. (2021) [36]	lvPPA: *n* = 41 nfvPPA: *n* = 44 svPPA: *n* = 62 HC: *n* = 60	Prospective group study	FU every year	(a) Cognition: ACE-III or ACE-R, WAIS-III (b) LOF: DAD	(a) Decline in overall cognition in all three variants but twice as rapid rate in lvPPA than nfvPPA and svPPA, Faster decline across the majority of cognitive domains in lvPPA. lvPPA: Worst performance on verbal fluency and memory domains at all time points. Attention and language higher at baseline but declined faster than all other subdomains. Greater decline than svPPA in memory and language subdomains but no difference with nfvPPA. nfvPPA: Disproportionate impairment in verbal fluency at all time points compared to other domains. Faster decline for language and memory. svPPA: Greater impairments in verbal fluency, language, and memory than other subdomains. (b) Faster rate of decline for lvPPA and nfvPPA compared to svPPA. Correlation between functional and cognitive decline for all groups across all time periods. Impact of cognition on functional capacity greater for nfvPPA at most time points.
Ulugut et al. (2021) [37]	lvPPA: *n* = 18 nfvPPA: *n* = 22 svPPA: *n* = 24	Retrospective group study	FU length 1 to 6 years	(a) Cognition: CDR, MMSE, RAVLT, FAB, VOSP, VAT (b) Language: BNT, VAT (c) BPSD: NPI (d) LOF: IADL	(a) lvPPA had more widespread cognitive deficits at baseline. Global cognitive decline in all groups overtime, especially svPPA and lvPPA. 83% of lvPPA acquired global cognitive impairment in line with the diagnostic criteria of dementia due to Alzheimer’s disease. (b) nfvPPA and lvPPA developed several additional language problems that met criteria for “PPA-extended” (other PPA syndrome). The majority of patients who showed mutism at FU were nfvPPA (7/8). (c) svPPA had more behavioural problems at baseline and at FU and 58% eventually met diagnostic criteria for bvFTD. (d) 65.6% met diagnostic criteria for “PPA-plus” and nfvPPA tended to develop motor deficits. 54% of nfvPPA eventually met criteria for CBS, PSP, or MND.

ACE-III = Addenbrooke’s Cognitive Examination III, ACE-R = Addenbrooke’s Cognitive Examination-Revised, AD = Alzheimer’s Disease, ADL = Activities of Daily Living, ApoE4 = Apolipoprotein E, BADL = Basic Activities of Daily Living, BDAE = Boston Diagnostic Aphasia Examination, BNT = Boston Naming Test, BPSD = Behavioural and Psychological Symptoms of Dementia, bvFTD = Behavioural Variant of Frontotemporal Dementia, CBD = Corticobasal Degeneration, CBI-R = Cambridge Behavioural Inventory Revised, CBS = Corticobasal Syndrome, CDR = Clinical Dementia Rating scale, DAD = Disability Assessment for Dementia, DRS = Mattis Dementia Rating Scale, FAB = Frontal Assessment Battery, FAQ = Functional Activities Questionnaire, FBI = Frontal Behavioural Inventory, FDS = Forward Digit Span, FTD = Frontotemporal Dementia, FTLD-CDR = Frontotemporal Lobar Degeneration-Clinical Dementia Rating Scale, FRS = Frontotemporal Dementia Rating Scale, FU = Follow-up, HC = Healthy Control, IADL = Instrumental Activities of Daily Living, IDDD = Interview for Deterioration in Daily Living Activities in Dementia, LOF = Level of Functioning, lvPPA = Logopenic Variant of Primary Progressive Aphasia, M = Mean, MMSE = Mini-Mental State Examination, MND = Motor Neuron Disease, NAT = Northwestern Anagram Test, nfvPPA = Non-Fluent Variant of Primary Progressive Aphasia, NPI = Neuropsychiatric Inventory, NPI-Q = Neuropsychiatric Inventory-Questionnaire, PASS = Progressive Aphasia Severity Scale, PPA = Primary Progressive Aphasia, PPVT = Peabody Picture Vocabulary Test, PSP = Progressive Supranuclear Palsy, RAVLT = Rey Auditory Verbal Learning Test, RCPM = Raven’s Coloured Progressive Matrices, RDS = Reverse Digit Span, ROC = Rate of Change, svPPA = Semantic Variant of Primary Progressive Aphasia, VAT = Visual Association Test, VOST = Visual Objective and Space Perception, WAB-AQ = Western Aphasia Battery Quotient, WAIS-III = Wechsler Adult Intelligence Scale-Third Edition, WAIS-R = Wechsler Adult Intelligence Scale Revised, WMS = Weschler Memory Scale.
